# Behavioural guidance of Chinook salmon smolts: the variable effects of LED spectral wavelength and strobing frequency

**DOI:** 10.1093/conphys/coy032

**Published:** 2018-06-25

**Authors:** Matthew J Hansen, Dennis E Cocherell, Steven J Cooke, Paul H Patrick, Michael Sills, Nann A Fangue

**Affiliations:** 1 Department of Wildlife, Fish and Conservation Biology, University of California, Davis, One Shields Ave, Davis, CA, USA; 2 Fish Ecology and Conservation Physiology Laboratory, Department of Biology, Institute of Environmental Science, Carleton University, 1125 Colonel By Drive, Ottawa, Ontario, Canada; 3 ATET-TECH, Inc., 68 Maxwell Court, Thornhill, Ontario, Canada

**Keywords:** Behavioural guidance, Chinook salmon, visual ecology, water diversion

## Abstract

Exploiting species-specific behavioural responses of fish to light is an increasingly promising technique to reduce the entrainment or impingement of fish that results from the diversion of water for human activities, such as hydropower or irrigation. Whilst there is some evidence that white light can be an effective deterrent for Chinook salmon smolts, the results have been mixed. There is a need to test the response of fish to different spectra and strobing frequencies to improve deterrent performance. We tested the movement and spatial response of groups of four fish to combinations of light-emitting diode (LED) spectra (red, green, blue and white light) during the day and night, and strobing frequencies (constant and 2Hz) during the day, using innovative LED technology intended as a behavioural guidance device for use in the field. Whilst strobing did not alter fish behaviour when compared to constant light, the red light had a repulsive effect during the day, with fish under this treatment spending significantly less time in the half of the arena closest to the behavioural guidance device compared to both the control and blue light. Importantly, this effect disappeared at night, where there were no differences in movement and space use found between spectra. There was some evidence of a potential attractive response of fish to the blue and green light during the day. Under these light treatments, fish spent the highest amount of time closest to the behavioural guidance device. Further tests manipulating the light intensity in the different spectra are needed to verify the mechanistic determinants of the observed behaviours. Results are discussed in reference to the known spectral sensitivities of the cone and rod photopigments in these fish, and further experiments are suggested to better relate the work to mitigating the effects on fish of infrastructure used for hydropower and irrigation.

## Teaser

We tested Chinook smolt response to combinations of spectra and strobing frequencies emitted from an underwater LED light, designed to guide fish near water diversion infrastructure. Whilst strobing did not alter fish behaviour when compared to constant light, the red light had a repulsive effect during the day, but not at night.

## Introduction

The disruption of freshwater river systems, resulting from hydropower infrastructure such as dams and water diversion for irrigation, can have negative effects on biodiversity and ecosystem functioning (Fahrig, 2003; Dudgeon *et al.*, 2006; Vorosmarty *et al.*, 2010). Chinook salmon (*Oncorhynchus tshawytscha*) are susceptible to water infrastructure where they can either get impinged against intake screens or forced into turbines. They are also susceptible to water diversions where fish are either impinged or entrained and transferred into machinery and irrigation ditches (Mussen *et al.*, 2013; 2015). These processes have been included as one of the reasons for population declines of Chinook salmon in California’s central valley, USA (Moyle *et al*., 2011). There is a continued need to create affordable and effective manipulations to water intake structures to reduce fish impingement and entrainment susceptibility without reducing the volume of water extracted and there is evidence that integrative techniques using sensory stimuli, in concert with physical barriers, can enhance fish protection systems (Nestler and Davidson, 1995; Ploskey *et al.*, 1998; Popper & Carlson, 1998; Sager *et al.*, 2000; Perry *et al.*, 2014; Ford *et al.*, 2017).

Research into the sensory ecology and conservation physiology of fishes has conceptualised the general idea of exploiting a fish’s innate behavioural response to visual, auditory or tactile environmental stimuli to distance themselves from harmful infrastructure; either by repelling fish from a dangerous path or directing them to a favourable path such as a bypass channel (Coutant, 1999; Noatch and Suski, 2012). White strobe lighting and mercury vapour bulbs are known to repel juvenile Chinook in large, low-velocity water bodies (Nemeth and Andersen, 1992; Brown, 2000; Mueller *et al.*, 2001; Johnson *et al.*, 2005; Richard *et al.,* 2007), however, there are mixed results. For example, differences in the light intensity can change the stimulus from being repulsive to attractive (Nemeth and Andersen, 1992), and the required power is a major implementation cost (Patrick *et al.*, 1985; Brown, 2000; Richards *et al.*, 2007). In river simulation conditions, where hydraulic conditions play a part, strobe lighting (4 × 200 lumens flashing white light-emitting diode (LED)) increased entrainment rates (Mussen *et al.*, 2014). Therefore, whilst there is some strong evidence that lighting can be used effectively for behavioural guidance (Brown, 2000; Nemeth and Andersen, 1992), there is specific need to test a range of light frequencies and to manipulate strobe frequency for improved performance.

The development of LED technology has allowed for simple, cheap and flexible programming of light stimuli at a variety of spectra and strobing frequencies. This is a useful tool as spectral sensitivity varies among species (Lythgoe, 1980). The spectra that a fish may be most sensitive to or find attractive or repulsive will be determined proximately by the types of photoreceptors in its retina (and the ratios of visual pigment within these photoreceptors). Ultimately, however, spectral sensitivity will be determined by the fish’s evolutionary history; including the influence of natural environmental light on prey detection and predator avoidance behaviour (Lythgoe, 1979; 1980; Munz and McFarland, 1977; Levine and MacNichol, 1979; Douglas and Hawyshyn, 1990). Sensitivity to certain wavelengths is likely an adaptation to the light environment that fish live in and is a good indication that these wavelengths are particularly useful for detecting objects in the water column (Lythgoe, 1979,^1980^; Munz and McFarland, 1977; Levine and MacNichol, 1982; Lythgoe and Partridge, 1989; Novales-Flamarique and Hawryshyn, 1993, 1994, 1997). However, spectral sensitivity does not necessarily lead to attractiveness or repulsiveness, and this can only be determined behaviourally.

Pacific salmon (*Oncorhynchus* sp.) have a well-developed broad-spectrum colour vision (Niwa and Tamura, 1969; Nakano *et al.*, 2006), but their spectral sensitivity changes as the ratio of visual pigments in their photoreceptors change throughout their life cycle (Beatty, 1966; Alexander, 1994; Novales-Flamarique, 2004, 2005; Nakano *et al.*, 2006; Temple *et al*., 2006) and in accordance to environmental stimuli (Beatty, 1966; Tsin and Beatty, 1977; Tsin, 1979; Allen and Munz, 1983; Cheng and Novales-Flamarique, 2004). For example, measures of visual pigment absorbance determined by microspectrophotometry in Chinook salmon smolts during March (60–80 days post-hatch (dph)) displayed max absorbance peaks of 430 nm in S-wave cones, 520 nm in M-wave cones, 560 nm in L-wave cones (all in bright light conditions) and 500 nm in rods (in dim light conditions) (Novales-Flamarique, 2005). These peaks shifted positively by May (100–140 dph), particularly in the L-wave cones, which increased by 40–600 nm (Novales-Flamarique, 2005). Using this information, in combination with other research on salmon vision (Beatty, 1966; Niwa and Tamura, 1969; Alexander *et al.*, 1994; Alexander, 1998; Parker and Hawryshyn, 2000; Hasegawa *et al.*, 2002; Allison *et al.*, 2006; Nakano *et al.*, 2006; Temple *et al.*, 2006; 2008), and work investigating the spectral sensitivities and behavioural guidance of other fish species (Hino, 1979; Furuse, 1999; Sillman *et al.*, 1995; Kawamura and Kishimoto, 2002; Sillman *et al.*, 2007; Sullivan *et al.*, 2016; Ford *et al.*, 2018), we have designed an experiment to test Chinook salmon smolt attraction and repulsion behaviour in controlled laboratory conditions.

We tested shoals of four fish as individuals of this species are rarely found in isolation in nature and are most likely to be in small schools at the life stage tested. Chinook salmon smolt spectral sensitivities (Parker and Hawryshyn, 2000; Novales-Flamarique, 2005) are similar to *Acipenser transmontanus*, which was found to be most attracted by green light and repulsed by red light (Ford *et al.*, 2018). Therefore, we tested green and red light, as well as blue and white light, as potential candidates for behavioural guidance of Chinook salmon smolts. We hypothesized that red will be most repulsive and green the most attractive during the day. We also tested the responses of smolts to different wavelengths of light at night because juvenile salmon often migrate during the night (Chapman *et al*., 2013). Juvenile Chinook salmon are primarily diurnal feeders (Sager and Glova, 1988; Schabetsberger, 2003) and may also likely avoid bright light at night as it potentially leaves them more exposed to predators (Yurk and Trites, 2000). Different visual systems are used in bright (photopic) and dim (scotopic) light conditions and different responses to white light have been found to occur across the photoperiod in salmon (Simmons *et al.*, 2004). At night, we hypothesize that the same spectra will be attractive (green) and repulsive (red), but that overall, fish will be more repulsed by light at night than during the day. The effect of an unnatural strobing of the light source is also predicted to increase the repulsive effects of light (Brown, 2000; Sager *et al*., 2000; Noatch and Suski, 2012; Ford *et al.*, 2018) and was tested at different spectra as fish can have different spectral sensitivities to ‘on’ and ‘off’ responses (Parker and Hawryshyn, 2000).

## Methods

Fish were acquired from the US Fish and Wildlife Service’s Coleman National Fish Hatchery (Anderson, California) in January 2017. Approximately 1000 fish were placed evenly into two 455-L flow through circular tanks supplied with groundwater from a well at UC Davis’ Center for Aquatic Biology and Aquaculture. Tanks were held outside in natural light conditions, with fine black mesh lids. Fish were held at 11°C for 3 months, before being raised to 16°C 3 weeks before experiments began. The fish were fed *ad libitum* commercial salmonid diet. Experiments were conducted in May 2017 when fish were ~150 dph. The mean ± SE fork length was 10.18 ± 0.11cm and the mean ± SE weight was 13.8 ± 0.46 g (*N* = 160).

Three experiments were conducted to test the behaviour of groups of four fish in response to combinations of spectra and strobing frequencies emitted from an underwater LED light. The light was developed by ATET-Tech, Inc. (Thornhill, ON) as a behavioural guidance device for migratory fishes, designed for use in a field setting. The device (35 × 12 × 9cm) consists of 162 LED modules that can each produce red, green and blue light and strobe at rates up to 40 Hz for all colour combinations.

The first two experiments consisted of five spectra treatments (blue, green, red, white light and a control treatment; where the behavioural guidance device was turned off (hereafter OFF)). Experiment 1 was conducted during the day from 9:00 to 14:00 h (6 May–10 May) and Experiment 2 was conducted during the night from 21:00 to 02:00h (15 May–19 May). We tested the fish at night (rather than in the dark during the day) as we wanted to capture any behavioural effects of circadian rhythm, not exclude them by keeping experimental time consistent. Experiment 3 consisted of three spectra treatments (blue, red and OFF) and two strobing frequencies (consistent and 2 Hz) and was conducted during the day from 09:00 to 14:00 h (23 May–26 May). Each day or night, two replicates of every treatment were conducted in a random order. Treatments in each experiment were repeated for a total of 10 times for Experiments 1 and 2, and nine times for Experiment 3, for a total of 616 fish used across the three experiments. All fish were naïve to the experimental conditions and only used once.

**Figure 1:**
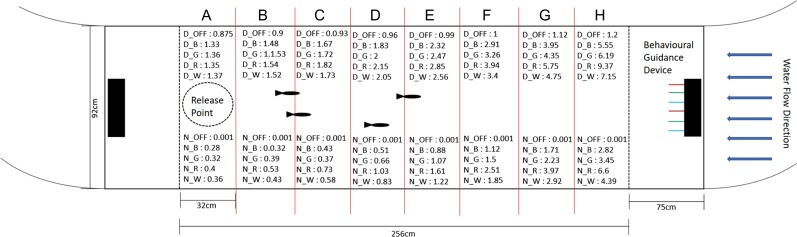
Overhead diagram of the arena showing dimensions, location of behavioural guidance device, release point, sections A–H and the quantum flux light readings (μmoles/m^2^/s) for day (D) and night (N) experiments. Blue arrows represent direction of water flow (0.15 m/s) inside the flume

Experiments were conducted within a 4000-L indoor flume set at a 0.15-m/s sweeping velocity to simulate a river current (Fig. 1). The test arena was a 92 cm × 256 cm section of the flume filled to a water depth of 30 cm. The two ends of the arena were sectioned off with stainless steel mesh wire to prevent fish escape. The floor of the arena was covered with black polyvinyl chloride (PVC) panels in Experiment 1 to reduce fish stress response (Barton, 2002). Pilot studies with Chinook salmon showed marked freezing and abnormal burst swimming behaviour in the arena with a white floor, presumably because the fish were too exposed in a novel environment. The floor of the arena was changed to dark grey PVC panels for the night experiments so that the fish were visible under infra-red floodlights (850 nm). Experiment 3 was also conducted with the dark grey PVC sheeting. For each experiment, the behavioural guidance device was placed at the upstream end of the arena, 55 cm in front of the mesh wire, and a model of the behavioural guidance device was placed at the downstream end (55 cm behind the mesh wire) as a visual control (Fig. 1). The entire experimental arena was surrounded by white curtains to reduce external disturbance and to ensure an even dispersion of background light.

At the beginning of an experimental trial, the behavioural guidance device was set to the required spectra and strobing frequency. Different coloured LEDs have different intensity outputs, such is the nature of LEDs. Red (longer wavelengths) have a greater photon density and intensity output than, for example, the blue LEDs (shorter wavelengths). The light intensity levels (μmoles/m^2^/s) within each section of the flume were therefore calculated using a quantum flux metre (LI-COR LI-1400) during the day, and during the night (Fig. 1). Four naive fish were then placed into a cylindrical cage created out of wire mesh (‘release point’ Fig. 1, diameter 45 cm) at the downstream end of the arena. After 5 min (Ford *et al.*, 2018), the cage was raised to 1 cm below the water surface using a rope and pulley system and fish were left to swim freely within the arena for 20 min. All trials were filmed and recorded using a Q-SEE security camera (4MP QTH8071B) mounted centrally 150 cm above the arena and connected to a DVR hard drive (Q-SEE QTH85) and an HD computer monitor. The arena was divided into eight sections (A–H), 32 cm apart. The section each fish was in was recorded every 30 s, giving 40-time points for each trial. These methods resemble other studies examining behavioural response to a light source (Marchesan *et al.*, 2005; Sullivan *et al.*, 2016). Also recorded was the number of times each fish crossed the halfway point of the arena (‘HW’), which was used as a measure of general activity. At the end of the 20-min trial, fish were netted and placed into a separate 455-L flow through tank. Ten trials were run consecutively each day, with each treatment replicated twice a day.

We performed a series of calculations to examine how spectra and strobing frequency affected the fish’s space use within the arena. To ensure independence of data points for analysis, for each time point in each trial, we summed the frequency of counts in Sections E through H and divided this by the total number of observations made during the trial (i.e. 160) to create a new variable ‘E-H,’ which is the proportion of time fish spent in the half of the arena closest to the light source, where light intensity of each spectra varied across the four sections (Fig. 1). We were also interested in how the fish responded to the highest light intensity of each spectrum, which occurred in Section H. Therefore, we ran the same calculations for section H alone and created a new variable ‘H’. As a measure of time fish spent in the E-H Section per visit (‘TEH’), we divided ‘E-H’ by the number of times fish crossed the halfway line of the arena (‘HW’). As the shading of the PVC flooring was not consistent between all experiments, the data was analysed separately for each experiment using analysis of variance (ANOVA), with light treatment as the independent variable and proportion of time in the E-H Section (‘E-H’), proportion of time in H Section (‘H’), general activity (‘HW’) or time per visit to the E-H Section (‘TEH’) as the dependent variable. Alpha was set at 0.05 and post-hoc assessments were Tukey-HSD tests. ‘E-H’ and ‘H’ were log transformed to meet assumptions of ANOVA. Assumptions of ANOVA could not be attained for the ‘HW’ or ‘TEH’ variables, in any of the three experiments, therefore they were analysed by Kruskall–Wallis *H* tests with Dunn tests used for post-hoc assessments. All analyses were conducted in R (v. 3.2.3, 2015).

## Results

**Figure 2:**
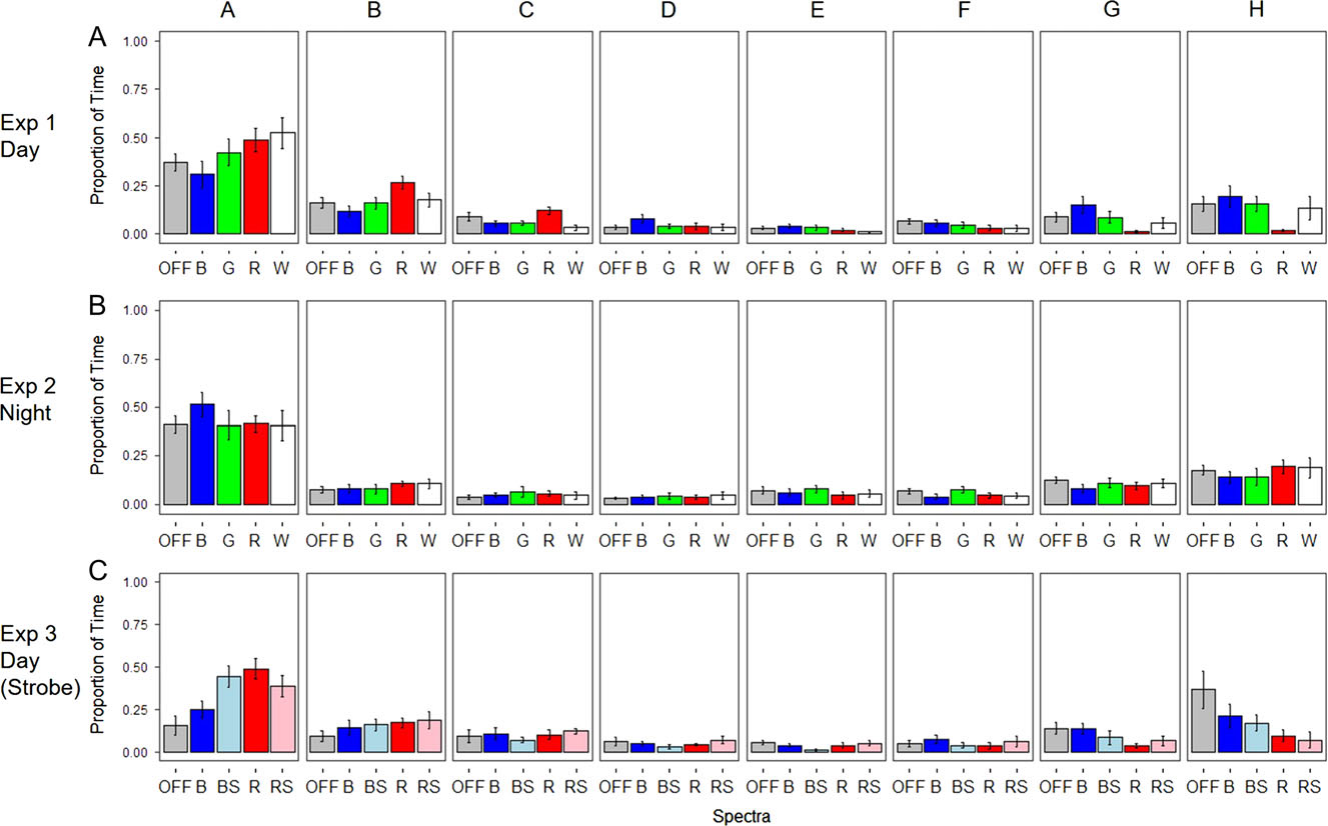
Bar graphs showing the proportion of time (*y*-axis) fish spent in each of the Sections (A–H) (*x*-axis) during each of the light treatments. The text below each *x*-axis and the colour of the bars represent the different spectra and strobing treatments emitted by the behavioural guidance device. ‘OFF’ = no light emitted, ‘B’ = Blue, ‘G’ = Green, ‘R’ = Red, ‘W’ = White, ‘BS’ = Blue and strobing at 2Hz, ‘RS’ = Red and strobing at 2Hz. Error bars represent SE

**Figure 3:**
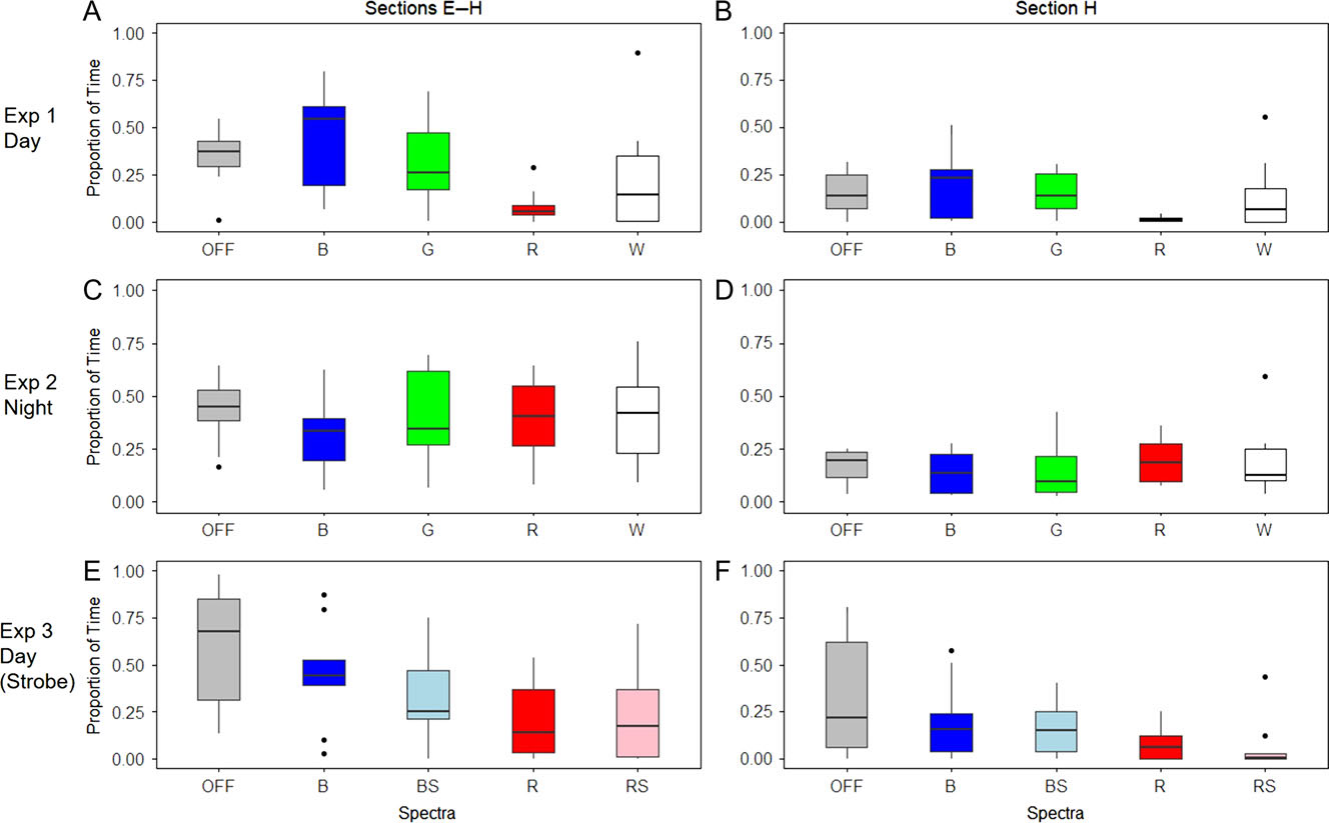
Boxplots of the proportion of time (*y*-axis) fish were in section E-H and H across each of the light treatments. The text below each *x*-axis and the colour of the bars represent the different spectra and strobing treatments emitted by the behavioural guidance device. ‘OFF’ = no light emitted, ‘B’ = Blue, ‘G’ = Green, ‘R’ = Red, ‘W’ = White, ‘BS’ = Blue and strobing at 2 Hz, ‘RS’ = Red and strobing at 2 Hz. Boxes represent first and third quartiles and whiskers extend to the highest value that is within 1.5 times the inter-quartile range.

**Figure 4:**
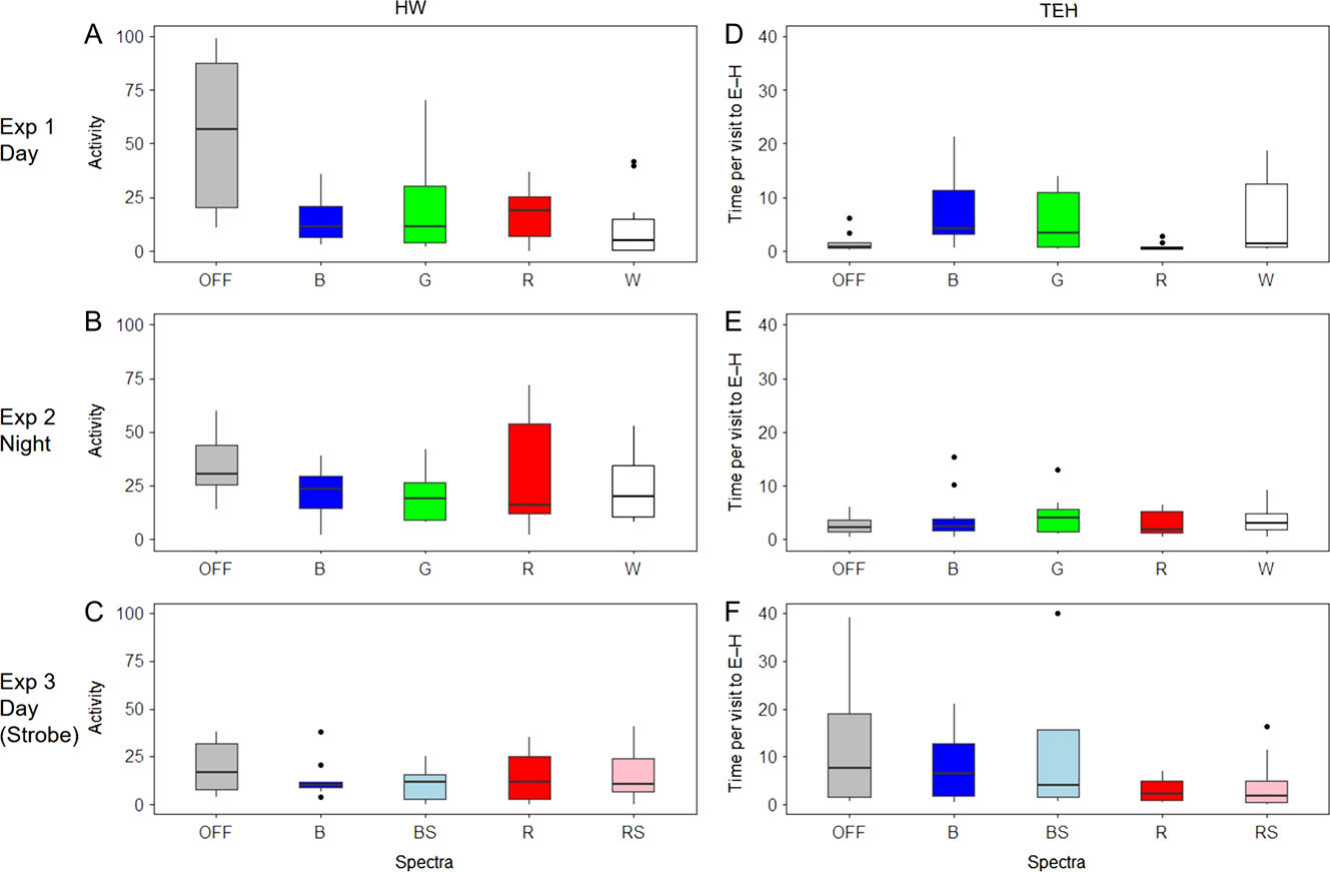
Boxplots of general activity (number of times fish passed the HW line) (*y*-axis) across each of the light treatments. Boxplots of time in Section E-H per visit (frequency of counts in Sections E–H divided by the number of times fish passed the halfway line) for each treatment. The text below each *x*-axis and the colour of the bars represent the different spectra and strobing treatments emitted by the behavioural guidance device. ‘OFF’ = no light emitted, ‘B’ = Blue, ‘G’ = Green, ‘R’ = Red, ‘W’ = White, ‘BS’ = Blue and strobing at 2 Hz, ‘RS’ = Red and strobing at 2 Hz. Boxes represent first and third quartiles and whiskers extend to the highest value that is within 1.5 times the inter-quartile range

In Experiment 1 (Day), spectral frequency had a significant effect on the proportion of time spent in the E-H Section (*F*_4,45_ = 4.099, *P* = 0.00645) (Figs 2a and 3a; Table 1), the proportion of time spent in Section H (*F*_4,45_ = 2.773, *P* = 0.0383) (Figs 2a and 3b; Table 1), on general activity (*H*_4_ = 12.358, *P* = 0.0148) (Fig. 4a; Table 1) as well as the amount of time spent in E-H per visit (*H*_4_ = 12.519, *P* = 0.0139) (Fig. 4d; Table 1). The smallest proportion of time spent in E-H and H was in the red treatment, and this was significantly different from both the OFF treatment (*P* = 0.046) and the blue treatment (*P* = 0.005) for Sections E-H (Figs 2a and 3a; Table 1), and the blue treatment for section H (*P* = 0.038) (Figs 2a and 3b; Table 1). Activity was highest in the OFF treatment (Fig. 4a; Table 1) which corresponded to fish in this treatment having a low amount of time in E-H per visit, although the shortest time in E-H per visit was with the red treatment (Fig. 4d; Table 1).

**Table 1:** Summary statistics for the proportion of time spent in Section E-H (‘E-H’), the proportion of time spent in Section H (‘H’), the number of times fish crossed the halfway line (general activity ‘HW’) and the time spent in Section E-H per visit (‘TEH’). The spectra treatments are: ‘OFF’ = no light emitted, ‘B’ = Blue, ‘G’ = Green, ‘R’ = Red, ‘W’ = White, ‘BS’ = Blue and strobing at 2Hz, ‘RS’ = Red and strobing at 2 Hz. Superscript letters represent significant differences between treatments within experiments

Experiment	Spectra	EH		H		TEH			HW		
		Mean	SE	Mean	SE	Median	Min	Max	Median	Min	Max
1-Day	OFF	0.34^b^	0.05	0.16^a,b^	0.04	0.74^b^	0.18	6.21	56.5^a,b^	11	99
	B	0.44^a,b^	0.08	0.19^b^	0.05	4.26^a,b^	0.52	21.2	11.5^b^	3	36
	G	0.32^a,b^	0.08	0.15^a,b^	0.04	3.42^a,b^	0.39	14	11.5^a,b^	2	70
	R	0.08^a^	0.03	0.02^a^	0.01	0.5^a,b^	0.25	2.88	19^b^	0	37
	W	0.23^a,b^	0.09	0.13^a,b^	0.06	1.4^a^	0.33	18.67	5^a,b^	0	42
2-Night	OFF	0.44	0.05	0.18	0.02	2.3	0.57	6.07	30.5	14	60
	B	0.32	0.05	0.14	0.03	2.47	0.41	15.5	23.5	2	39
	G	0.41	0.07	0.14	0.04	3.99	1.1	13	19	8	42
	R	0.38	0.06	0.19	0.03	1.89	0.57	6.5	27	2	126
	W	0.40	0.07	0.19	0.05	3.15	0.53	9.2	20	8	53
3-Day Strobe	OFF	0.60^b^	0.11	0.36^b^	0.11	7.71	0.76	39	17	4	38
	B	0.45^a,b^	0.09	0.21^a,b^	0.07	6.6	0.56	21	10	4	38
	BS	0.30^a,b^	0.08	0.17^a,b^	0.05	4	0.72	40	12	0	25
	R	0.20^a^	0.06	0.09^a,b^	0.03	2.36	0.5	7	12	0	35
	RS	0.24^a^	0.09	0.07^a^	0.05	1.89	0	16.29	11	0	41

In Experiment 2 (Night), spectra frequency had no effect on either the proportion of time spent in E-H Section (*F*_4,45_ = 0.542, *P* = 0.706) (Figs 2b and 3c; Table 1) or Section H (*F*_4,45_ = 0.563, *P* = 0.69) (Figs 2b and 3d; Table 1), general activity (*H*_4_ = 1.4942, *P* = 0.8277) (Fig. 4b; Table 1), or the amount of time spent in E-H per visit (*H*_4_ = 1.4248, *P* = 0.839) (Fig. 4e, Table 1).

In Experiment 3 (Strobe), spectra frequency had a significant effect on the proportion of time spent in E-H Section (*F*_4,45_ = 3.327, *P* = 0.019) (Figs 2c and 3e; Table 1) and Section H (*F*_4,45_ = 2.806, *P* = 0.038) (Figs 2c and 3f; Table 1) but neither on general activity (*H*_4_ = 5.1731, *P* = 0.27) (Fig. 4c; Table 1) nor the amount of time spent in E-H per visit (*H*_4_ = 4.3292, *P* = 0.363) (Fig. 4f; Table 1). The smallest proportion of time spent in E-H was in the red treatment and the red strobe treatment, and these were both significantly different from the OFF treatment (*P* = 0.022 and *P* = 0.047, respectively) (Figs 2c and 3e; Table 1). For Section H, red strobe treatment was significantly different from the OFF treatment (*P* = 0.03) (Figs 2c and 3f, Table 1).

## Discussion

Our data show that the behavioural response of Chinook salmon smolts to an LED behavioural guidance device depends on both the spectra emitted and the time of day. The most notable discovery was that the red light during the day had a moderate repulsive effect, with fish spending 26% less time in the half of the arena closest to the light source compared with the control OFF treatment. No colour had an attractive effect. Chinook salmon have developed colour vision to ensure their vision can function effectively under a wide range of environmental conditions (Munz and McFarland, 1977; Levine and MacNichol, 1979, 1982), and are known to have photoreceptors sensitive to all spectral wavelengths tested (Novales-Flamarique, 2005). Fish, however, was repulsed by the red light specifically and not the other spectra. The maximum rod (500 nm) and cone (520 nm) spectral sensitivities of Chinook smolts (dph 83) (Novales-Flamarique, 2005) closely match the green, middle wavelength dominated background light environment of the Pacific coastal and river systems (Novales-Flamarique and Hawryshyn, 1993). This ‘sensitivity hypothesis’ is thought to allow predators to optimally detect prey items against background light (Munz and McFarland, 1977; Lythgoe, 1979; 1980; Lythgoe and Partridge, 1989). For objects brighter than the background light (e.g. the behavioural guidance device), better contrast is attained if the fish’s wavelength sensitivity is ‘offset’ from the background light environment (Lythgoe and Partridge, 1989). Therefore, for wild Chinook smolts swimming in relatively dim, greenish background light, a bright red light may stand out better than a bright green light, even though their maximum spectral sensitivity is in the middle wavelengths.

Crucially, however, the background light environment of the flume during the day was white, not the green of their natural environment, so it is less likely that the red light contrasting best with the background light explains the differences found between spectra, as all spectra would have been equally offset to the background light. Still, there may be an inherent sensitivity to red, because under natural conditions it contrasts best with background light. The spectral sensitivity of various salmonids (including *Onchorynchus sp.*) under white light background conditions has been examined. The OFF response (voltage change in the optic nerve after a ‘decrement’ of a light stimulus) is dominated by middle-wavelength green-light sensitivity, however, the ON-response (voltage change in the optic nerve after an ‘increment’ of a light stimulus) is dominated by long-wavelength red-light sensitivity (Coughlin and Hawryshyn, 1994a, 1994b; Novales-Flamarique and Hawryshyn, 1997; Parker and Hawryshyn, 2000).

Maximal spectral sensitivity may therefore not be a perfect predictor of the behavioural response to a light source in this species. However, it is possible that an ON-response sensitivity to longer wave lengths under white-light background conditions played a part in determining the behavioural response of Chinook salmon smolts to the red light treatment. The red light had the highest light intensities (Fig. 1) compared to the other spectra (especially the blue and the green) which likely influenced results (Nemeth and Andersen, 1992). The effect of white light, which had the next highest light intensity, was intermediary between red light and blue and green light. We tested the brightest LED output from the behavioural guidance device for each spectral frequency, as these intensities are of practical use for large-scale use of the device in the field. The red light from the LEDs inherently has a greater photon density than the other spectral frequencies, which means it was not possible with the current data to tease apart spectral frequency and light intensity. Closer examination of the light intensities emitted by each colour in the different sections does suggest that it is not intensity alone that determines repulsiveness, as the photon density of the red light in Section G is less than or equal to that of the green and blue light in Section H, and yet the red light was still repulsive. There are spatial confounds with this type of examination and our results to date are merely suggestive. More stringent tests are needed in the future to adequately describe the precise mechanisms determining the behaviours observed. The light intensity of the different spectra may enhance their potential repulsiveness or, particularly in the case of blues and greens, their potential attractiveness (Nemeth and Andersen, 1992). Future tests will have to specifically vary light intensity with spectral frequency so that, for example, reds and blues of the same light intensity can be compared to verify both the effect of colour, intensity and diurnal differences in avoidance behaviour, especially in relation to red light.

In a closely related species, *O. masou masou*, differences in spectra were not found to have any influence in attempts to behaviourally guide the fish with light (Terazono *et al.*, 1998) nor were there differences found between light treatments for behavioural guidance of *Micropterus salmoides* (Sullivan *et al.*, 2016) despite the fact that this species is known to have spectral sensitivity to red wavelengths (Kawamura and Kishimoto, 2002). Maximal spectral sensitivity, however, has been a good predictor of behavioural response in other species, with *Plecoglossus altivelis*, effectively frightened by red light (570 nm), which is close to its peak spectral sensitivity (Hino, 1979; Furuse, 1999) and *A. transmontanus* found to be most attracted to green light which most closely matched their peak spectral sensitivities (Ford *et al.*, 2018). Similar to Chinook salmon and *P. altivelis*, *A. transmontanus* were repulsed by red light in well-lit conditions, as were the marine *Lithognathus mormyrus* and the euryhaline *Mugil cephalus* (Marchesan *et al.*, 2005). Unfortunately, these papers did not report light intensity levels (μmoles/m^2^/s) of the different colours they tested, and therefore we cannot comment on what role any inherent differences in light intensity of the different spectral frequencies may have played in these experiments.

Juvenile Chinook salmon are primarily diurnal feeders (Sager and Glova, 1988; Schabetsberger, 2003) and the lack of response to different spectra at night may be related to their feeding motivation. However, activity levels generally increased at night in this study compared to during the day, except for in the OFF treatment, where light level was so low that any visual stimulus may have been absent. In our night experiment, fish likely perceived the darker conditions to be safer to move in. This latter idea is supported by our observation that there was less of a ‘U-shaped’ distribution to the spatial data at night (see Fig. 2a and b), and fish spent more time in the middle sections (e.g. Section E), which would naturally have led them to cross the HW line more often. Activity and space use were generally different for fish during Experiment 3 compared with Experiment 1, most noticeably there was a drop in activity in the OFF treatment, and fish spent more time upstream in the E–H Sections in all treatments. It is not entirely clear why there was a change in movement behaviour between the two-day experiments as fish were always naive and external conditions such as flow rate, lighting and time of day were consistent between the experiments. Additional replication may have cleared up the differences between experiments. The only variable that changed was the shading of the flooring, which was dark grey rather than black, and it is intuitive to suggest fish felt safer to explore more on the darker background. However, it is very encouraging that the repulsive effects of the red light were robust to a behavioural change between the two separate experiments and to the slight differences in the shading of the floor.

During Experiment 1, under the blue and green-light treatments, fish had the greatest amount of time spent in E–H per visit (Fig. 4d), suggesting these spectra engaged the attention of the fish. However, further testing is needed to confirm these wavelengths were attractive under these conditions or whether it was the specific light intensity emitted. Indeed, in Experiment 3, where fish did not show very high levels of exploratory activity in the OFF treatment, there was no significant difference between the OFF or blue treatment in either the proportion of time spent in the E–H Sections (‘E–H’), or the time spent in E–H per visit (‘TEH’). However, the blue light was intermediate to the consistently repulsive red treatment.

The strobing effect (2 Hz) of the blue and the red light did not differentially affect fish behaviour, with red strobe having a repulsive effect like the constant red light and with the blue strobe having a similar effect to the constant blue light. There was a small amount of evidence that the strobing red light may have a stronger repulsive effect than the constant red light if one only considers the time spent in the section closest to the light source (‘H’). However, differences were minimal and more tests are needed. The strobing rate in our experiment was quite low at 2 Hz and this rate should have been discernable by juvenile Chinook salmon (Johnson *et al.*, 2005). Red strobing magnified the repulsive effect of red light during the day in *A*. t*ransmontanus* (Ford *et al.*, 2018). It is known that strobing white light can be effective for Chinook behavioural guidance (Johnson *et al.*, 2005, although see Mussen *et al.*, 2014), therefore it is not surprising the red strobe treatment had an effect considering other *Oncorhynchus* sp. of similar size (*Oncorhynchus mykiss* and *Oncorhynchus c clarki*) are known to have high red light sensitivity of the ON-response (Coughlin and Hawryshyn, 1994a, 1994b; Parker and Hawryshyn, 2000).

Whilst this study provides some evidence that light spectra may influence behavioural guidance of Chinook salmon smolts, continued experiments are necessary. This is particularly true if behavioural guidance of different aged Chinook is needed, since the ratio of their photopigments changes throughout their life history (Beatty, 1966; Alexander *et al.*, 1994; Alexander, 1998; Hasegawa *et al.*, 2002; Cheng and Novales Flamarique, 2004; Allison *et al.*, 2006; Temple *et al.*, 2006, 2008) suggesting that their behavioural response to different wavelengths also changes during ontogeny. Future work must concentrate on manipulating light intensity as well as spectral frequency, and should include evaluation of finer scale differences in movement (speed, turning speed, intermittent locomotion characteristics) and shoaling behaviour (inter-individual distances and shoal position).

Animal movement is strongly influenced by an individual’s motivation, but it is the interaction with external environmental conditions that ultimately determines where an animal moves (Nathan *et al.*, 2008). A flow velocity of 0.15 m/s was used to simulate a river current, and fish were started downstream as they showed positive rheotaxis, but from a fish management perspective (hydropower or irrigation), it is necessary to conduct future tests with fish starting upstream of the behavioural guidance device and at higher flows up to 0.3 m/s. Therefore, we suggest that further tests should occur *in situ* or in larger flumes that more accurately represent the hydraulic conditions near water diversion infrastructure (e.g. Perry *et al.,* 2014; Mussen *et al.*, 2013, 2014). The reason for this is that responses of fish to flow can dominate their behavioural decisions, limiting or downgrading their response to other behavioural stimuli (Patrick *et al.*, 1985; Carlson, 1994; Popper and Carlson, 1998; Enders *et al.*, 2009). Future experiments should also consider the effects of temperature (due to its effect on exploration and activity rates as well as photopigment ratio (Allen and McFarland, 1973; McFarland and Allen, 1977; Tsin and Beatty, 1977; Alexander *et al.*, 1994)), as well as turbidity. Increased particulate matter (‘gelbstoff’: shortwave absorbing compounds) in turbid waters causes substantial changes in light intensity and spectral frequency, shifting background light into longer wavelengths (Levine and MacNichol, 1979; Utne-Palm, 2002). What produces an effect in some hydraulic or environmental conditions may not be effective in others, and we need a systematic assessment of fish response to light stimuli for combinations of environmental factors that are manipulated along gradients.

In conclusion, we have built on the body of work examining light as a behavioural guidance device for migrating Chinook salmon smolts by testing the effect of a light-emitting behavioural guidance device on their movement and space use in a laboratory setting. Whilst there was some evidence that the blue and the green light may attract the attention of Chinook smolts during the day, there was no effect of different spectra during the night and more studies manipulating light intensity alongside spectral frequency are needed. Strobing a spectrum at 2 Hz made no significant difference. The primary result, of interest to those studying basic visual ecology and behaviour of *Onchorynchus* sp., as well as those in fisheries management, is that the red light from the behavioural guidance device had a moderate repulsive effect during the day but not during the night.
